# Gabapentin for chronic pelvic pain in women (GaPP2): a multicentre, randomised, double-blind, placebo-controlled trial

**DOI:** 10.1016/S0140-6736(20)31693-7

**Published:** 2020-09-26

**Authors:** Andrew W Horne, Katy Vincent, Catherine A Hewitt, Lee J Middleton, Magda Koscielniak, Wojciech Szubert, Ann M Doust, Jane P Daniels, GaPP2 collaborative, Suraiya Abdi, Santanu Acharya, Shamma Al-Inizi, Elizabeth Ball, Andrew Baranowski, Nadia Bhal, Kalsang Bhatia, Siladitya Bhattacharya, Judy V. Birch, Tyrone Carpenter, Tony Chalhoub, Ying C. Cheong, T. Justin Clark, Roman Cregg, Tunde D. Dada, Dib Datta, Radwan Faraj, Max G. Feltham, Pratima Gupta, Dharani Hapangama, Chris Hardwick, Jon Hughes, Pinky Khatri, Geeta Kumar, Lisa J. Leighton, Kingshuk Majumder, Gary J. McFarlane, Alex Mortimer, Smita Odedra, Bruce Ramsay, Amer Raza, William Rea, Somendra Roy, Afia Sajid, Lucky Saraswat, Ahmar Shah, Jambulingam Sivasamy, Rashmi Srivastava, Clive Stubbs, Ajay Swaminathan, Premila Thampi, Omar Thanoon, Tony Thomas, Irene Tracey, Martyn Underwood, Clare Willocks, Amanda C de C. Williams, Simon Wood, Krina Zondervan

**Affiliations:** a MRC Centre for Reproductive Health, Queen's Medical Research Institute, University of Edinburgh, Edinburgh, UK; b Nuffield Department of Women's and Reproductive Health, University of Oxford, Oxford, UK; c Birmingham Clinical Trials Unit, University of Birmingham, Birmingham, UK; d Nottingham Clinical Trials Unit, University of Nottingham, Nottingham, UK

## Abstract

**Background:**

Chronic pelvic pain affects 2–24% of women worldwide and evidence for medical treatments is scarce. Gabapentin is effective in treating some chronic pain conditions. We aimed to measure the efficacy and safety of gabapentin in women with chronic pelvic pain and no obvious pelvic pathology.

**Methods:**

We performed a multicentre, randomised, double-blind, placebo-controlled randomised trial in 39 UK hospital centres. Eligible participants were women with chronic pelvic pain (with or without dysmenorrhoea or dyspareunia) of at least 3 months duration. Inclusion criteria were 18–50 years of age, use or willingness to use contraception to avoid pregnancy, and no obvious pelvic pathology at laparoscopy, which must have taken place at least 2 weeks before consent but less than 36 months previously. Participants were randomly assigned in a 1:1 ratio to receive gabapentin (titrated to a maximum dose of 2700 mg daily) or matching placebo for 16 weeks. The online randomisation system minimised allocations by presence or absence of dysmenorrhoea, psychological distress, current use of hormonal contraceptives, and hospital centre. The appearance, route, and administration of the assigned intervention were identical in both groups. Patients, clinicians, and research staff were unaware of the trial group assignments throughout the trial. Participants were unmasked once they had provided all outcome data at week 16–17, or sooner if a serious adverse event requiring knowledge of the study drug occurred. The dual primary outcome measures were worst and average pain scores assessed separately on a numerical rating scale in weeks 13–16 after randomisation, in the intention-to-treat population. Self-reported adverse events were assessed according to intention-to-treat principles. This trial is registered with the ISRCTN registry, ISCRTN77451762.

**Findings:**

Participants were screened between Nov 30, 2015, and March 6, 2019, and 306 were randomly assigned (153 to gabapentin and 153 to placebo). There were no significant between-group differences in both worst and average numerical rating scale (NRS) pain scores at 13–16 weeks after randomisation. The mean worst NRS pain score was 7·1 (standard deviation [SD] 2·6) in the gabapentin group and 7·4 (SD 2·2) in the placebo group. Mean change from baseline was −1·4 (SD 2·3) in the gabapentin group and −1·2 (SD 2·1) in the placebo group (adjusted mean difference −0·20 [97·5% CI −0·81 to 0·42]; p=0·47). The mean average NRS pain score was 4·3 (SD 2·3) in the gabapentin group and 4·5 (SD 2·2) in the placebo group. Mean change from baseline was −1·1 (SD 2·0) in the gabapentin group and −0·9 (SD 1·8) in the placebo group (adjusted mean difference −0·18 [97·5% CI −0·71 to 0·35]; p=0·45). More women had a serious adverse event in the gabapentin group than in the placebo group (10 [7%] of 153 in the gabapentin group compared with 3 [2%] of 153 in the placebo group; p=0·04). Dizziness, drowsiness, and visual disturbances were more common in the gabapentin group.

**Interpretation:**

This study was adequately powered, but treatment with gabapentin did not result in significantly lower pain scores in women with chronic pelvic pain, and was associated with higher rates of side-effects than placebo. Given the increasing reports of abuse and evidence of potential harms associated with gabapentin use, it is important that clinicians consider alternative treatment options to off-label gabapentin for the management of chronic pelvic pain and no obvious pelvic pathology.

**Funding:**

National Institute for Health Research.

## Introduction

Chronic pelvic pain is estimated to affect 2–24% of women worldwide, and is associated with substantially reduced quality of life and a 45% reduction in work productivity.^1^, ^2^, ^3^ It can be associated with underlying pathology such as endometriosis, but in up to 55% of women, no obvious cause is identified at laparoscopy.^3^ Management of chronic pelvic pain within gynaecological practice is difficult as no established treatments are available, but careful exploration of symptoms and history can point to non-gynaecological causes of chronic pelvic pain, for which some effective treatments exist.^4^

**Research in context**

**Evidence before this study**

At the time of the design of the pilot study for this trial (GaPP1), there had been an increase in prescription of gabapentin in general within the UK. The rate of patients newly treated with gabapentinoids in primary care tripled from 2007 to 2017, and by 2017 half of the prescriptions of gabapentinoids were for an off-label indication. At the time, we also observed that there was considerable use of gabapentin for chronic pelvic pain (largely because of its perceived effectiveness in other chronic pain conditions). First, we surveyed a random group of general practitioners, with the support of the Scottish Primary Care Research Network. Of the general practitioners who responded to our survey, 74% said that they would consider gabapentin as a treatment option for chronic pelvic pain in women. Second, we surveyed a random group of gynaecologists, with the support of the Royal College of Obstetricians and Gynaecologists. Of those who responded to our survey, 50% said that they currently prescribe gabapentin for chronic pelvic pain, and 92% said that they would consider gabapentin as a treatment option for this condition. Since then, awareness and use of gabapentinoids has continued to increase in gynaecology, with the publication of reviews in this area and reference within the National Institute for Health and Care Excellence (NICE) guideline for endometriosis to the NICE guideline on neuropathic pain for treatment of chronic pelvic pain with neuromodulators. However, data from randomised clinical trials of the use of gabapentin in women with chronic pelvic pain are scarce. A 2017 Cochrane review identified one trial that compared the efficacy of gabapentin and amitriptyline for chronic pelvic pain in women with a range of pelvic pathologies, but this study was open-label, there was no placebo group, the population had a mixed aetiology of pain symptoms, and the numbers analysed were small, with only 56 participants. An update to the Cochrane review search strategy in MEDLINE, Embase, PsychLit, and CAB abstracts to April 15, 2020, found only our own pilot trial (GaPP1) and one other placebo-controlled trial from Egypt, neither of which were powered to detect significant differences, and both had substantial attrition. An increased risk of suicidal behaviour, as a potential side-effect of gabapentin, and possible misuse of the drug have been of concern with the rise in the prescribing of gabapentin.

**Added value of this study**

This study is the first large, randomised, placebo-controlled clinical trial to report on treatment of chronic pelvic pain with gabapentin. Although it conflicts with the results of the Egyptian trial, the robustness of the study design, including masking to treatment allocation of both participants and investigators, ensured internal validity, enabling the results to be interpreted with confidence. Groups were balanced with respect to dysmenorrhoea, psychological distress, and concomitant use of hormonal contraceptives—all potentially prognostic for reported pain. The design of our trial reflects the real-word choices that women and their gynaecologists make about the management of chronic pelvic pain. We can confidently conclude that gabapentin is not effective for chronic pelvic pain in women.

**Implications of all the available evidence**

Women with chronic pelvic pain and no obvious pelvic pathology should be advised that gabapentin might not alleviate their pain and could give them unpleasant side-effects. In our opinion, no further research is required to establish the role of gabapentin in the management of chronic pelvic pain in women with no obvious pelvic pathology. Questions that remain unaddressed relate to the use of other pharmacological interventions (monotherapy *vs* combination therapy), physiotherapy, and cognitive behavioural therapy for treating chronic pelvic pain in women. It is possible that subgroups of women could benefit from gabapentin, but these will need to be carefully characterised and explored.

The off-label use of gabapentin for chronic pelvic pain has increased because of its proven efficacy in other chronic pain conditions.^5^, ^6^ Gabapentin primarily affects modulation of pain by the CNS, and neuroimaging studies have shown gabapentinoids affect brain function in models of central sensitisation and in patients with chronic pain.^7^, ^8^ There is also some evidence for peripheral activity.

Data from randomised clinical trials of the use of gabapentin in women with chronic pelvic pain are scarce. One trial compared the efficacy of gabapentin and amitriptyline for chronic pelvic pain in women with a range of pelvic pathologies^9^ but this study was open-label, there was no placebo group, the population had a mixed aetiology of pain symptoms, and the numbers analysed were small with only 56 participants in total. GaPP1, our own pilot trial of gabapentin, was not powered to detect meaningful differences between gabapentin and placebo for chronic pelvic pain.^10^ Another randomised, placebo-controlled trial of 60 women did show a (statistically, and potentially clinically) significant difference in patient-reported pain after 12 weeks of treatment but the variability of patient responses was considerably lower than all previous studies in chronic pelvic pain, and not generalisable outside of that population.^11^ In this study, we aimed to establish the efficacy and safety of gabapentin in women with chronic pelvic pain and no obvious pelvic pathology.

## Methods

### Study design and participants

This multicentre, randomised, double-blind, placebo-controlled trial (GaPP2) took place in 39 UK hospital centres. Ethical approval for the trial was obtained from the UK Coventry and Warwick Research Ethics Committee (REC 15/WM/0036) and clinical trial authorisation was obtained from the Medicines and Healthcare Products Regulatory Authority. A trial steering committee provided independent oversight of the trial. Confidential interim analysis of all available data alongside anonymised reports of adverse events by participants were reviewed by a data monitoring committee on four occasions. No reason to recommend halting or modifying the trial was identified. The trial protocol has been published elsewhere.^12^

The recruitment criteria for this study were defined before the International Classification of Diseases-11 classification for chronic secondary visceral pain was drawn up, and were based on the Royal College of Obstetricians and Gynaecologists definition for chronic pelvic pain and the 2012 International Association for the Study of Pain taxonomy.^13^, ^14^ Moreover, we believe that they reflect the group of women commonly seen in gynaecological practice within the UK and that any heterogeneity is a strength, ensuring that our results are generalisable. Eligible participants were women with chronic pelvic pain (with or without dysmenorrhoea or dyspareunia) of at least 3 months duration. We limited the study to women because of the growing body of evidence from preclinical and clinical studies that suggest sex differences in pain thresholds, sensitivity, and underlying neuroimmune modulation.^15^, ^16^ Pelvic pain was defined as pain located within the true pelvis (between and below the anterior iliac crests). Inclusion criteria were 18–50 years of age, use or willingness to use contraception to avoid pregnancy, and no obvious pelvic pathology at laparoscopy (eg, macroscopic endometriosis lesions, complex ovarian cysts or ovarian cysts of >5 cm, fibroids of >3 cm, and dense adhesions), which must have taken place at least 2 weeks before consent but less than 36 months previously. Women were excluded if they only had dysmenorrhoea; had a malignancy; were currently using or had previously used gabapentin or pregabalin; had surgery planned in next 6 months; had contraindications to taking gabapentin; had a previous reaction to gabapentin; were taking gonadotrophin-releasing hormone agonists and were unable or unwilling to stop; were breastfeeding, pregnant, or planned a pregnancy in next 6 months; or had pain suspected to be of gastrointestinal origin (according to positive Rome III Diagnostic Criteria); or had previously participated in the GaPP1 pilot study. All women provided written informed consent. After confirmation of clinical eligibility, women entered a pre-randomisation screening phase where they were required to return their worst and average pain scores on a numerical rating scale (NRS) ranging from 0 (no pain) to 10 (worst pain imaginable), weekly for 4 weeks via a centralised text messaging system. To be considered eligible for randomisation, women were required to have returned at least three of four pre-randomisation NRS scores on both the worst and average NRS scales to show adherence to data collection. At least two of the worst NRS pain scores needed to be a score of 4 or higher, for the pain to be considered sufficient for entry into the trial. No study drugs were taken during this pre-randomisation screening phase, but participants were able to remain on any analgesics they were taking. We did not specify a target recruitment per centre; all centres recruited as many women as they could during the recruitment period.

### Randomisation and masking

Participants were randomly assigned in a 1:1 ratio to receive either gabapentin or matched placebo through a secure online randomisation system, with the use of minimisation to balance trial group assignments according to presence or absence of dysmenorrhoea (pain score of ≥4 on a 0–10 NRS), psychological distress (General Health Questionnaire^17^ score ≥2), current use of hormonal contraceptives, and hospital centre. The appearance, route, and administration of the assigned intervention were identical in both groups. Patients, clinicians, and research staff were unaware of the trial group assignments throughout the trial. Participants were unmasked once they had provided all outcome data at week 16–17, or sooner if a serious adverse event requiring knowledge of the study drug occurred.

### Procedures

Participants took the assigned drugs orally, every day from the time of randomisation to 16 weeks after randomisation. The study drugs were supplied by Sharp Clinical Services UK, who procured the gabapentin and manufactured the placebo capsule, over-encapsulated the gabapentin, and dispensed all capsules into numbered containers. The dosing regimen included a 4-week titration phase where each participant started on one capsule (300 mg) daily, and increased the dose by one capsule every 3 days until they perceived that they were gaining adequate pain relief, or reported side-effects that precluded them from further increases, up to a maximum dose of nine capsules. If necessary, participants were advised to titrate down to the last tolerated dose with minimal side-effects. They were also asked to maintain their highest tolerated dose until the end of week 16. Study drugs were dispensed at time of randomisation, at weeks 4–5, and (dependent on the maximum tolerated dose reached) at weeks 8–10. Other analgesic medications, including opioids and antidepressants (but excluding those contraindicated alongside gabapentin) were allowed and their use was recorded.

### Outcomes

The dual primary outcome measures of worst and average pain scores were recorded on a NRS at 13–16 weeks after randomisation. They were collected via weekly text messages and assessed or interpreted as separate outcomes. The final worst pain score was taken as the worst response from the worst pain scores returned, and the average pain score as the mean of the average pain scores returned. Non-responders were contacted by telephone and the pain scores reported verbally. Secondary outcome measures comprised an examination at 16 weeks after randomisation of the proportion of women who had a 30% or 50% reduction in worst and average NRS pain scores (from baseline to end of treatment); global patient impression of change; general quality of life (Short Form-12),^18^ further pain assessment (Brief Pain Inventory),^19^ assessment for neuropathic-like features of pain (PainDETECT),^20^ fatigue assessment (Brief Fatigue Inventory),^21^ psychological distress (General Health Questionnaire),^17^ pain-related cognitions (Pain Catastrophizing Scale),^22^ impairments in paid work or activities (Work and Productivity Activity Impairment Questionnaire),^23^ and sexual functioning (Sexual Activity Questionnaire).^24^ Patient-reported questionnaires were collected at weeks 16–17 post-randomisation. The Pelvic Pain and Urinary Frequency Patient Symptom Scale was completed at baseline only. Data on use of analgesics, serious adverse events, and side-effects were collected at weeks 4–5 and 8–10, either when the study drug was dispensed or by telephone. Women were asked to report appointments with health-care professionals (eg, general practitioner or hospital doctor). Reporting was restricted to appointments in relation to chronic pelvic pain, in addition to those required by the trial. We attempted to collect outcome data for all participants who were randomly assigned, regardless of adherence to the trial group assignment. Adherence to the study drug was collected using treatment diaries which recorded the number of tablets taken daily, alongside self-reported estimates of adherence. Adherence to treatment was defined as taking more than 50% of the study drug, self-reported at weeks 16–17.

### Statistical analysis

The planned sample size of 240 women was estimated to provide 90% power to detect a minimally important clinical difference in NRS scores of one point on a 0–10 scale, assuming a SD of 2·5. There is a body of literature^10^, ^25^ to support one point on a NRS or 1 cm on a visual analogue scale representing minimal or little change, and 2·0–2·7 points being much or some change. To account for any increase in the risk of type 1 error that could be associated with having dual outcome measures, a Bonferroni correction was applied (with a two-sided α-level of 0·025). We planned to include 300 women in the trial to account for up to 20% loss to follow-up.

The dual primary outcome measures are presented as means and SDs, alongside adjusted mean differences (with corresponding 97·5% CIs) produced from a linear regression model. Mean differences were adjusted for baseline score and minimisation variables. Mean change from baseline is also presented by group. The primary analysis is equivalent to a change from baseline analysis when also adjusting for baseline score. 97·5% CIs have been used and p values have been reported from two-sided tests at the 2·5% significance level, to account for the Bonferroni correction applied to the dual primary outcome measures. NRS scores were further analysed using a repeated-measures model in which all assessment times were included. Binary secondary outcomes are presented as risk ratios produced from a log-binomial regression model. All continuous secondary outcome measures are presented as mean differences produced from a linear regression model. Appointments with health-care professionals for chronic pelvic pain and use of analgesics, in addition to appointments required by the trial, are reported descriptively only. All secondary outcomes are reported as point estimates with 99% CIs (without p values). All analyses, including assessment of safety and adverse events, were done by intention to treat and adjusted for the presence of dysmenorrhoea, psychological distress defined by the General Health Questionnaire,^17^ current use of hormonal contraceptive, recruiting hospital, and baseline score. Sensitivity analyses for the dual primary outcomes include a per-protocol analysis, an assessment of missing primary outcome data by means of a multiple-imputation approach, and an analysis to assess the effect of time between screening and randomisation.

Three prespecified subgroup analyses were done on the basis of presence or absence of dysmenorrhoea, psychological distress defined by the General Health Questionnaire^17^ (score 0–1 or 2–12), and current use of hormonal contraceptives (yes or no). These subgroup analyses were limited to the dual primary outcomes only. The treatment effect within these subgroups was examined by the measurement of treatment-by-subgroup interaction in the linear regression model.

Interim analyses of effectiveness and safety endpoints were done by the trial statistician (who remained unaware of treatment assignments) on behalf of the data and safety monitoring committee at approximately 12-month intervals during the recruitment period. Because the interim analyses were done according to the Haybittle–Peto principle,^26^ no adjustment was made in the final p values to measure significance. All analyses were done in SAS (version 9.4). This trial is registered with the ISRCTN registry, ISCRTN77451762.

### Role of the funding source

The funder of the study had no role in study design, data collection, data analysis, data interpretation, writing of the report, or decision to submit the results for publication. The corresponding author and trial statisticians had full access to all the data in the study. All authors in the writing team shared final responsibility for the decision to submit for publication. The manufacturers of the gabapentin drug used for this trial and Sharp Clinical Services UK were not involved in any aspect of the study.

## Results

**Figure 1:**
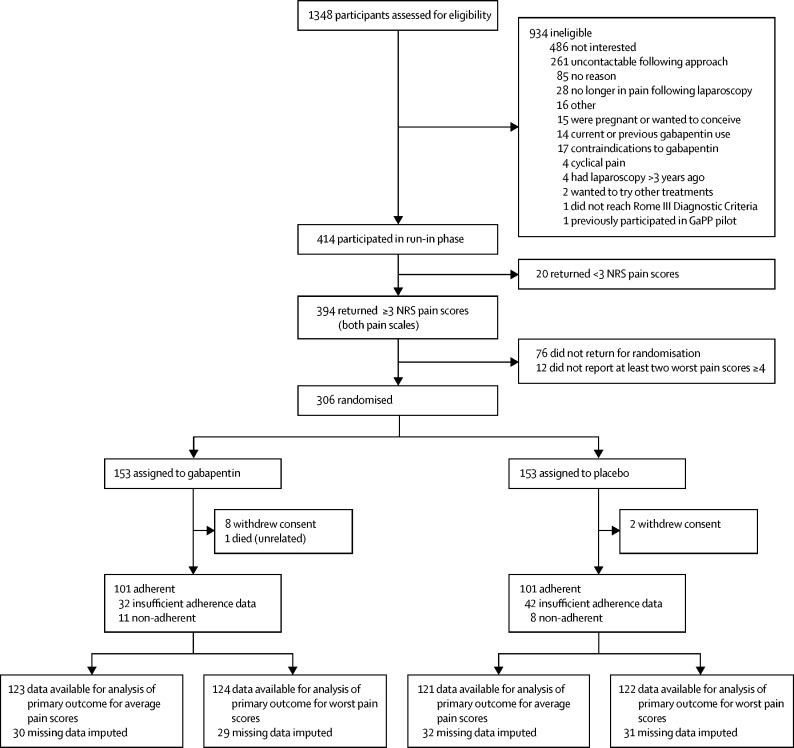
Trial profile The final worst pain score was taken as the worst response from the worst pain scores returned, and the average pain score as the mean of the average pain scores returned. Adherence to treatment was defined as taking more than 50% of the study drug, self-reported at weeks 16–17. Participants were withdrawn from any further follow-up if they withdrew consent. NRS=numerical rating scale.

Participants were screened between Nov 30, 2015, and March 6, 2019. Of the 1348 women who were approached for participation, 414 were initially considered eligible based on clinical criteria (figure 1). Of these 414 women, 306 were randomly assigned (153 to gabapentin and 153 to placebo). 80% of participants had available data for the dual primary outcome measures (246 [80%] worst scores returned; 244 [80%] average scores returned). The groups were well balanced for all characteristics measured at baseline (table 1).

**Table 1:** Demographic and baseline characteristics of participants

		**Gabapentin (n=153)**	**Placebo (n=153)**
Dysmenorrhoea*		100 (65%)	100 (65%)
GHQ score for anxiety and depression†		38 (25%)	38 (25%)
GHQ total score†		4·6 (3·7)	4·7 (3·7)
Current use of sex hormones		99 (65%)	99 (65%)
	Patch	2/99 (2%)	0
	Combined oral contraceptive pill	26/99 (26%)	21/99 (21%)
	Progesterone-only pill	19/99 (19%)	16/99 (16%)
	Levonorgestrel-releasing intrauterine system	38/99 (38%)	45/99 (45%)
	Implant	12/99 (12%)	12/99 (12%)
	Injection	5/99 (5%)	8/99 (8%)
Age (years)		30·5 (7·7)	30·1 (8·6)
Ethnicity			
	White	150 (98%)	148 (97%)
	Black (Caribbean, African, or other)	1 (1%)	0
	Asian (Indian, Pakistani, Bangladeshi, or other)	2 (1%)	4 (2%)
	Mixed (Caribbean, African, Asian, or other)	0	1 (1%)
Body-mass index (kg/m^2^)		27·1 (5·7), 151	27·8 (5·9), 150
Education			
	Primary	4 (3%)	5 (3%)
	Secondary	47 (31%)	46 (30%)
	Tertiary	101 (66%)	101 (66%)
	Missing	1 (1%)	1 (1%)
Menstruating		109 (71%)	108 (71%)
Pain score during periods*		7·7 (1·6), 103	7·6 (1·7), 103
PUF symptom score‡		9·7 (4·1)	10·0 (4·5), 148
PUF bother score‡		5·3 (2·6)	5·4 (2·8), 150
PUF total score‡		15·0 (6·3)	15·5 (7·0), 147
Rescue medications		114 (75%)	112 (73%)
	Non-steroidal anti-inflammatory drugs	62/114 (54%)	66/112 (59%)
	Opiates	78/114 (68%)	68/112 (61%)
	Other (includes paracetamol)	61/114 (54%)	58/112 (52%)
Neuropathic pain§		32 (21%)	33 (22%)
Missing¶		1	4

Data are n (%); mean (SD); mean (SD), N; or n/N (%) when N is different to the total number of participants. GHQ=General Health Questionnaire. PUF=Pelvic Pain and Urinary Frequency Patient Symptom Scale.

* Dysmenorrhoea is defined as a pain score ≥4 during periods. Pain scores range from 0 to 10, where 0 is no pain and 10 is the worst pain imaginable.

† GHQ scores range from 0 to 12, where higher scores represent higher amounts of mental distress. A score of 0 or 1 meets the definition of anxiety and depression.

‡ PUF symptom score ranges from 0 to 23, PUF bother score ranges from 0 to 12, and PUF total score ranges from 0 to 35; a score greater than 12 is indicative of clinically significant symptoms.

§ Defined as a PainDETECT score ≥19.

¶ Participants missing a neuropathic pain (PainDETECT) score.

**Figure 2:**
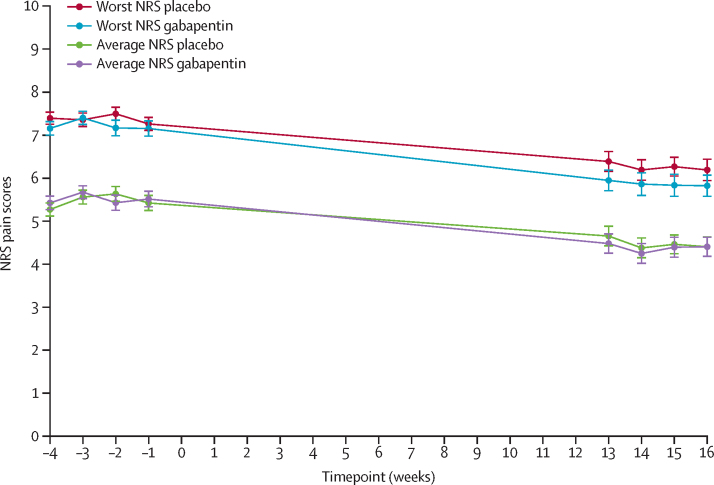
Longitudinal plot of primary outcome measurements in each trial group Error bars indicate the SE. NRS=numerical rating scale

There were no significant between-group differences in both worst and average NRS pain scores at 13–16 weeks after randomisation. The mean worst NRS pain score was 7·1 (SD 2·6) in the gabapentin group and 7·4 (SD 2·2) in the placebo group. Mean change from baseline was −1·4 (SD 2·3) in the gabapentin group and −1·2 (SD 2·1) in the placebo group (adjusted mean difference −0·20 [97·5% CI −0·81 to 0·42]; p=0·47). The mean average NRS pain score was 4·3 (SD 2·3) in the gabapentin group and 4·5 (SD 2·2) in the placebo group. Mean change from baseline was −1·1 (SD 2·0) in the gabapentin group and −0·9 (SD 1·8) in the placebo group (adjusted mean difference −0·18 [97·5% CI −0·71 to 0·35]; p=0·45; table 2; figure 2).

**Table 2:** Primary outcomes

	**Baseline**		**End of study**		**Change from baseline**		Mean difference*(97·5% CI; p value)
	Gabapentin (n=153)	Placebo (n=153)	Gabapentin (n=153)	Placebo (n=153)	Gabapentin	Placebo	
Worst NRS pain score	8·4 (1·3)	8·6 (1·2)	7·1 (2·6), 124	7·4 (2·2), 122	−1·4 (2·3), 124	−1·2 (2·1), 122	−0·20 (−0·81 to 0·42; p=0·47)
Average NRS pain score	5·5 (1·7)	5·5 (1·7)	4·3 (2·3), 123	4·5 (2·2), 121	−1·1 (2·0), 123	−0·9 (1·8), 121	−0·18 (−0·71 to 0·35; p=0·45)

Data are mean (SD) or mean (SD), N when N is different to the total number of participants, unless otherwise specified. NRS=numerical rating scale.

* Adjusted for baseline score and minimisation variables. Values <0 favour gabapentin. Threshold for significance α=0·025 because of Bonferroni correction.

Point estimates and CIs from the prespecified sensitivity analyses and further analyses from the repeated-measures model were consistent with the primary analysis (appendix pp 2–3). There was no evidence of varying effect in the three prespecified subgroup analyses (appendix p 4).

No significant differences were noted in the proportion of women who had a reduction in NRS scores from baseline or any other patient-reported secondary outcomes (table 3). Women in the gabapentin group reported they were taking fewer painkillers, but these differences were not significant (appendix p 5).

**Table 3:** Secondary outcomes

	**Baseline**		**End of study**		**Estimate (99% CI)**
	Gabapentin (n=153)	Placebo (n=153)	Gabapentin (n=153)	Placebo (n=153)	
**Reduction in NRS score from baseline (≥30%)**					
Worst NRS pain score	..	..	30/124 (24%)	21/122 (17%)	1·38 (0·72 to 2·64)*
Average NRS pain score	..	..	44/123 (36%)	37/121 (31%)	1·12 (0·70 to 1·80)*
**Reduction in NRS score from baseline (≥50%)**					
Worst NRS pain score	..	..	19/124 (15%)	10/122 (8%)	1·84 (0·71 to 4·75)*
Average NRS pain score	..	..	27/123 (22%)	19/121 (16%)	1·36 (0·68 to 2·72)*
**Patient global impression of change**					
Very marked or marked improvement	..	..	34/112 (30%)	22/108 (20%)	1·48 (0·80 to 2·73)†
Minimal improvement or worsening‡	..	..	78/112 (70%)	86/112 (80%)	..
**Patient-reported questionnaires**					
Short Form-12 mental component score	40·3 (10·8)	39·5 (11·3), 149	41·3 (10·6), 111	42·5 (11·1), 110	−1·11 (−4·60 to 2·39)§
Short Form-12 physical component score	39·0 (9·2)	40·1 (9·4), 149	43·8 (10·6), 111	44·6 (10·1), 110	0·49 (−2·27 to 3·24)§
Brief Pain Inventory pain interference score	4·9 (2·6), 152	5·0 (2·6), 152	3·6 (2·8), 111	3·6 (2·8), 112	−0·04 (−0·84 to 0·77)¶
Brief Fatigue Inventory global fatigue score	5·3 (2·4)	5·1 (2·3), 152	4·2 (2·5), 111	4·0 (2·7), 112	0·12 (−0·65 to 0·89)¶
General Health Questionnaire total score	4·6 (3·7)	4·7 (3·7)	3·8 (3·9), 111	3·0 (3·5), 111	0·72 (−0·49 to 1·94)¶
WPAIQ activity impairment score	53·4 (25·1)	52·1 (25·4), 151	39·3 (29·0), 110	38·6 (29·6), 111	−0·77 (−9·66 to 8·12)¶
WPAIQ absenteeism score‖	10·9 (23·2), 117	12·0 (25·6), 121	10·8 (23·5), 83	4·9 (15·1), 89	5·32 (−2·06 to 12·71)¶
WPAIQ presenteeism score**	47·1 (26·2), 109	46·5 (26·7), 104	36·4 (28·4), 72	38·0 (29·6), 79	−1·89 (−14·43 to 10·65)¶
WPAIQ work productivity loss score**	49·7 (27·9), 109	49·2 (28·2), 103	39·9 (31·1), 72	39·2 (30·7), 79	−0·43 (−13·73 to 12·87)¶
Pain Catastrophising Questionnaire total score	27·4 (12·9)	27·2 (13·0), 152	20·8 (14·6), 111	19·7 (12·5), 111	0·48 (−3·24 to 4·20)¶
SAQ pleasure score††	10·2 (4·1), 117	9·7 (4·8), 101	10·8 (4·5), 83	10·9 (4·1), 69	−0·14 (−1·84 to 1·56)§
SAQ discomfort score††	2·9 (1·6), 117	3·1 (1·8), 100	3·6 (1·9), 84	3·3 (2·0), 68	0·17 (−0·55 to 0·90)§
SAQ habit score††	0·8 (0·6), 118	0·6 (0·6), 101	1·1 (0·8), 83	0·9 (0·7), 69	0·19 (−0·15 to 0·53)§
PainDETECT total score	13·6 (6·9), 152	13·3 (6·5), 149	12·4 (6·8), 111	10·9 (6·7), 107	1·19 (−0·74 to 3·12)¶

Data are n/N (%); mean (SD); or mean (SD), N, when N is different to the total number of participants, unless otherwise specified. Patient-reported questionnaires were collected at weeks 16–17 post-randomisation. Worst and average NRS, Brief Pain Inventory, and Brief Fatigue Inventory scores range from 0 to 10, Short-Form 12 and WPAIQ scores range from 0 to 100, General Health Questionnaire scores range from 0 to 12, Pain Catastrophising Questionnaire scores range from 0 to 52, SAQ pleasure scores range from 0 to 18, SAQ discomfort scores range from 0 to 6, SAQ habit scores range from 0 to 3, and PainDETECT scores range from −1 to 38. NRS=numerical rating scale. WPAIQ=Work Productivity and Activity Impairment Questionnaire. SAQ=Sexual Activity Questionnaire.

* Risk ratio (99% CI), adjusted for baseline score and minimisation variables. Values >1 favour gabapentin.

† Risk ratio (99% CI), adjusted for minimisation variables.

‡ Includes minimal improvement, no change, minimal worsening, marked worsening and very marked worsening. Values >1 favour gabapentin.

§ Mean difference (99% CI), adjusted for baseline score and minimisation variables. Values >0 favour gabapentin.

¶ Mean difference (99% CI), adjusted for baseline score and minimisation variables. Values <0 favour gabapentin.

‖ In women who are currently employed.

** In women who are currently employed and working >0 hours in the past 7 days.

†† In women who are currently sexually active (baseline: gabapentin=123, placebo=105; week 16: gabapentin=87, placebo=74); the table reports only data for those who are sexually active who returned data.

A higher proportion of women had a serious adverse event in the gabapentin group (10 [7%] of 153) than in the placebo group (3 [2%] of 153; p=0·04; table 4). One participant who was on gabapentin died of a complication of pneumonia which was exacerbated by other comorbidities, but this was not considered related to study participation. Known side-effects (as reported in the summary of product characteristics) of dizziness, drowsiness, and visual disturbances were more common in the gabapentin group than in the placebo group (table 4), but did not appear to prompt greater use of health-care resources (appendix p 7).

**Table 4:** Summary of reported side-effects and adverse events

	**Gabapentin (n=153)**	**Placebo (n=153)**	Risk ratio*(99% CI)	**p value**
**Side-effects**				
Dizzy	66/122 (54%)	32/114 (28%)	1·91 (1·22–2·99)	0·0002
Tired	85/129 (66%)	68/120 (57%)	1·12 (0·86–1·44)	0·3
Drowsy	64/124 (52%)	34/116 (29%)	1·71 (1·09–2·68)	0·002
Change in mood	55/118 (47%)	43/112 (38%)	1·17 (0·79–1·74)	0·3
Change in urinary pattern	37/114 (32%)	35/111 (32%)	1·00 (0·61–1·63)	1·0
Visual disturbances	25/113 (22%)	12/110 (11%)	2·25 (0·99–5·10)	0·01
Change in skin	31/112 (28%)	23/110 (21%)	1·35 (0·74–2·50)	0·2
Different pain	33/116 (28%)	37/117 (32%)	0·88 (0·53–1·46)	0·5
Shortness of breath	17/114 (15%)	11/109 (10%)	1·45 (0·57–3·71)	0·3
**Adverse events**				
Serious adverse event	10/153 (7%)	3/153 (2%)	..	0·04

Data are n/N (%), unless otherwise specified. The total number of serious adverse events was 15 (12 in the gabapentin group, and three in the placebo group).

* Adjusted for baseline score and minimisation variables. Values <1 favour gabapentin.

Adherence to the study drug was similar between both the gabapentin and placebo groups (101 [90%] in the gabapentin group compared with 101 [93%] in the placebo group). A detailed breakdown of the extent of self-reported adherence to the study drug is shown in the appendix (p 8). The overall median dose taken daily per week was calculated for each group and is presented in the appendix (p 9). The doses used during the escalation phase were equal between the groups, thereafter the participants in the gabapentin group generally took one capsule (300 mg) more than those in the placebo group throughout the treatment period. At week 4, the median maximum tolerated number of tablets was equivalent to 2100 mg in both groups, although this reached 2700 mg (maximum permitted dose) in future weeks for some women.

## Discussion

This multicentre, randomised, double-blind, placebo-controlled trial showed that, in women with chronic pelvic pain and no obvious pelvic pathology, gabapentin was no more effective than placebo in reducing pain. The trial was powered adequately to detect differences between trial groups, and the between-group point estimates and corresponding CIs excluded a minimally important clinical difference of one point on a 0–10 pain scale, so we can confidently conclude that gabapentin is not effective for chronic pelvic pain at a group level. The incidence of side-effects and serious adverse events was higher in the gabapentin group than in the placebo group. We do not believe that the inclusion of a small proportion of women with neuropathic-like pain (65 [22%] of 301 participants) and potentially a number of actual neuropathies, explains the lack of efficacy of gabapentin over placebo overall in our study, given that there was no difference in mean PainDETECT scores between the two groups at baseline nor at the end of the study.

We used a dual primary outcome measure and considered both worst and average pain scores. These outcomes were considered separately and an improvement in one (or both) would conclude gabapentin was efficacious. Although we acknowledge that it is preferable to have a single primary outcome, a survey of our patient involvement group found that worst and average pain were equally important to women.^26^ We therefore collected the pain scores weekly over a 4-week period, asking participants to rate both worst and average pain for the preceding week, and defined a minimum number of responses to create a valid outcome. We used this approach because chronic pelvic pain can fluctuate during a woman's menstrual cycle, so eliciting a pain score at a single timepoint is unlikely to capture the effect of gabapentin nor reflect the woman's experience of pain. All outcome data in the trial were subjective or participant-reported outcomes (rather than laboratory measurements), but the study was blinded, reducing risk of incurring assessor bias.

The calculation for the sample size for the trial was based on a recognised minimally important clinical difference for chronic pain^25^ of one point on a 0–10 NRS, and used a SD from a comparable pilot study.^10^ Appropriate adjustments were applied to account for the dual primary outcome in both the sample size calculation and analysis. The target number of women were recruited and missing outcome data were as anticipated, with a follow-up rate for the dual primary outcome of 80% of women (246 [80%] worst NRS and 244 [80%] average NRS). The sensitivity analysis was almost identical to the observed data comparison and the CIs for both did not reach the minimally important clinical difference, so it is unlikely a meaningful treatment effect was missed because of missing data.

Rate of adherence to the trial regimen was high (women reported taking at least half of the study drug doses throughout the trial), but was not validated against an objective method such as pill-counting. The dose of gabapentin that participants received was based on individual adjustment of the dose by the participants themselves, which reflected their perception of pain relief and side-effects. Adjustments were made in accordance with existing dosing recommendations,^27^ up to a dose of 2700 mg per day, and final doses ranged from 600 mg to 2700 mg per day during the treatment phase. When the trial protocol was written, 2700 mg was the maximum dose recommended by the British National Formulary, and only pain clinics used to prescribe above this dose. We recognise that it is now generally accepted that higher doses can be prescribed, if side-effects permit.

We also acknowledge that it would have been interesting to look at the effect of review by a pain specialist or specialist pain medicine prescription on outcomes, but we did not document whether patients had been seen by a pain specialist at the outset of the trial. However, it is worth noting that within the UK, pain management approaches for chronic pelvic pain are not usually considered until after all gynaecological and other relevant specialist (eg, urology, gastroenterology) assessments and appropriate treatments are complete, or not providing symptom improvement. Therefore, it is unlikely that the women enrolled in this trial would have been seen by a pain specialist with regards to their pelvic pain.

Although more women in the placebo group were able to correctly guess their allocation at the end of the treatment period (78 [74%] of 106 correctly guessed placebo and 64 [58%] of 111 correctly guessed gabapentin), their use of rescue medication was similar. We cannot conclude that women who perceived they were taking placebo compensated by increasing their analgesic use and thus negated any effect of gabapentin.

The higher rates of side-effects observed with gabapentin compared with placebo (eg, dizziness, drowsiness, and visual disturbances) in the trial are consistent with other published studies. A 2018 meta-analysis of all trials for postherpetic neuralgia and painful diabetic neuropathy^28^ showed that, compared with placebo, gabapentin was associated with more drowsiness (gabapentin 14% *vs* placebo 5%; p<0·001) and dizziness (gabapentin 19% *vs* placebo 7%; p<0·001). Nevertheless, the rates of side-effects in our trial are lower than in these other studies, despite only measuring these in women. This would suggest that women are not more susceptible to developing side-effects from gabapentin than men.

Similar to many clinical trials for treatments for pain, we observed a potential placebo effect.^29^ The trial was not designed to investigate the neurobiological mechanisms behind this effect, but information offered in relation to treatment, patients' expectations, previous encounters with gabapentin, and the therapeutic milieu can all generate this response.^30^ Nonetheless, the placebo effect observed is very relevant, because of the side-effect profile of gabapentin and its potential addictive properties.^31^

In conclusion, our results show that gabapentin did not relieve pain in women with chronic pelvic pain, and that gabapentin was associated with higher rates of side-effects than placebo.

## Data sharing

Requests for data should be directed to the lead author (andrew.horne@ed.ac.uk). Patient-level data will be made available within 6 months of publication. Requests will be assessed for scientific rigour before being granted. Data will be anonymised and securely transferred. A data sharing agreement may be required.
