# Department of Error

**DOI:** 10.1016/S0140-6736(20)32159-0

**Published:** 2020-10-24

*Horne AW, Vincent K, Hewitt CA, et al. Gabapentin for chronic pelvic pain in women (GaPP2): a multicentre, randomised, double-blind, placebo-controlled trial.* Lancet *2020; **396:** 909–17*—In this Article, the final three references were omitted from the reference list. This correction has been made to the online version as of Oct 22, 2020.

