# Supplementary material for: Gabapentin for chronic pelvic pain in women (GaPP2): a multicentre, randomised, double-blind, placebo-controlled trial
Source: Lancet. 2020 Sep 26;396(10255):909–17. doi: 10.1016/S0140-6736(20)31693-7 (PMC7527829; doi:10.1016/S0140-6736(20)31693-7)

THE LANCET

Supplementary appendix

This appendix formed part of the original submission and has been peer reviewed. We post it as supplied by the authors.

Supplement to: Horne AW, Vincent K, Hewitt CA, et al. Gabapentin for chronic pelvic pain in women (GaPP2): a multicentre, randomised, double-blind, placebo-controlled trial. *Lancet* 2020; **396:** 909–17.

Supplementary Material

Table S 1 Sensitivity analyses of primary outcome

	Baseline		End of study[X]			
	**Gabapentin** Mean (SD, n)	**Placebo** Mean (SD, n)	**Gabapentin** Mean (SD, n)	**Placebo** Mean (SD, n)	**Mean Difference[2]** (97·5% CI)	**p-value[3]**
**Worst NRS pain scores[1]**						
Per-protocol analysis*	8.5 (1·1, 101)	8·6 (1·2, 101)	7·3 (2·3, 98)	7·5 (2·2, 97)	-0·14 (-0·81, 0·53)	0·6[3]
Multiple imputation for missing data	-	-	-	-	-0·19 (-0·72, 0·33)	0·5[3]
Effect of time between screening and randomization	8·4 (1·3, 153)	8·6 (1·2, 153)	7·1 (2·6, 124)	7·4 (2·2, 122)	-0·20 (-0·82, 0·42)	0·5[3]
**Average NRS pain scores[1]**						
Per-protocol analysis*	5·6 (1.6, 101)	5·4 (1.7, 101)	4·4 (2·0, 98)	4·5 (2·1, 97)	-0·23 (-0·81, 0·35)	0·4[3]
Multiple imputation for missing data	-	-	-	-	-0·21 (-0.66, 0.24)	0·4[3]
Effect of time between screening and randomization	5·5 (1·7, 153)	5·5 (1·7, 153)	4·3 (2·3, 123)	4·5 (2·2, 121)	-0·18 (-0·71, 0·35)	0·4[3]

[X] Worst and average NRS pain scores measures weeks 13-16 post-randomization.
[1] Pain scores range from 0 to 10, where 0=no pain and 10=worst pain imaginable.
[2] Adjusted for baseline score and minimization variables (including time between screening and randomization for effect of time between screening and randomization sensitivity analysis only). Values <0 favour gabapentin.
[3] Threshold for statistical significance α=0·025 due to Bonferroni correction.
* The per-protocol cohort only includes those adherent with treatment allocation (gabapentin=101, placebo=101).

Table S 2 Repeated measures analysis

	**Gabapentin** Mean (SD, N)	**Placebo** Mean (SD, N)	**Interaction p-value**	**Mean Difference**[2] (97.5% CI)
Worst NRS pain scores[1]			0·8	-0·29 (-0·87, 0·29)
Week 13	6·0 (2·7, 123)	6·4 (2·5, 115)		
Week 14	5·9 (2·9, 119)	6·2 (2·6, 118)		
Week 15	5·8 (2·8, 118)	6·3 (2·4, 118)		
Week 16	5·8 (2·6, 116)	6·2 (2·7, 118)		
Average NRS pain scores[1]			0.8	-0.11 (-0·62, 0·41)
Week 13	4·5 (2·5, 122)	4·7 (2·4, 114)		
Week 14	4·3 (2·5, 118)	4·4 (2·5, 115)		
Week 15	4·4 (2·5, 118)	4·5 (2·3, 118)		
Week 16	4·4 (2·4, 115)	4·4 (2·4, 117)		

[1]Pain scores range from 0 to 10, where 0=No pain and 10=Worst pain imaginable.

[2]Adjusted for baseline score and minimization variables. Values <0 favour Gabapentin.

Note: Average NRS was taken as the mean pain score across weeks 13-16, worst NRS was taken as the worst response (highest score) across weeks 13-16.

Table S 3 Subgroup analysis of primary outcome

	**Gabapentin** Mean (SD, n)	**Placebo** Mean (SD, n)	**Interaction p-value**
**Worst NRS pain scores[1]**			
Dysmenorrhea			0·7
Yes	7·2 (2·3, 79)	7·4 (2·3, 78)	
No	6·8 (2·9, 45)	7·3 (2·2, 44)	
GHQ score			0·4
0-1	6·5 (2·4, 35)	6·6 (2·2, 33)	
2-12	7·3 (2·6, 89)	7·6 (2·2, 89)	
Use of sex hormones			0·1
Yes	7·2 (2·5, 81)	7·1 (2·3, 81)	
No	6·8 (2·7, 43)	8·0 (2·0, 41)	
**Average NRS pain scores[1]**			
Dysmenorrhea			0·3
Yes	4·5 (2·1, 79)	4·4 (2·2, 77)	
No	3·9 (2·5, 44)	4·6 (2·2, 44)	
GHQ score			0·1
0-1	3·8 (2·2, 35)	3·6 (2·2, 33)	
2-12	4·5 (2·3, 88)	4·8 (2·1, 88)	
Use of sex hormones			0·3
Yes	4·5 (2·3, 81)	4·3 (2·1, 81)	
No	3·9 (2·1, 42)	4·8 (2·3, 40)	

[1]Pain scores range from 0 to 10, where 0= No pain and 10=Worst pain imaginable.

Table S 4 Summary of use of painkillers

	**Week 4-5**		**Week 8-10**		**Week 16-17**[1]	
	Gabapentin	Placebo	Gabapentin	Placebo	Gabapentin	Placebo
	(N=118)	(N=121)	(N=111)	(N=108)	(N=103)	(N=101)
Use of painkillers since taking study medication *n (%)*						
Less	65 (55)	61 (50)	57 (51)	52 (48)	52 (50)	42 (42)
Same	42 (36)	46 (38)	38 (34)	42 (39)	40 (39)	45 (44)
More	11 (9)	14 (12)	16 (15)	14 (13)	11 (11)	14 (14)

[1]Weeks 16-17 is the end of study assessment.

Table S 5 Rescue medication

	Baseline		Week 4-5		Week 8-10		Week 16-17[1]	
	**Gabapentin**	**Placebo**	**Gabapentin**	**Placebo**	**Gabapentin**	**Placebo**	**Gabapentin**	**Placebo**
N[2]	(N=153)<br>n (%)	(N=153)<br>n (%)	(N=136)<br>n (%)	(N=138)<br>n (%)	(N=119)<br>n (%)	(N=119)<br>n (%)	(N=112)<br>n (%)	(N=109)<br>n (%)
Rescue Medication[3]	114 (75%)	112 (73%)	68 (50%)	70 (51%)	66 (55%)	58 (49%)	62 (55%)	64 (59%)
NSAIDS[4]	62/114 (54%)	66/112 (59%)	31/68 (46%)	36/70 (51%)	26/66 (39%)	27/58 (47%)	25/62 (40%)	29/64 (45%)
Opiates[5]	78/114 (68%)	68/112 (61%)	33/68 (49%)	34/70 (49%)	38/66 (58%)	31/58 (53%)	34/62 (55%)	27/64 (42%)
Other[6]	61/114 (54%)	58/112 (52%)	39/68 (57%)	34/70 (49%)	38/66 (58%)	31/58 (53%)	34/62 (55%)	31/64 (48%)

[1]Weeks 16-17 is the end of study assessment.
[2]Attended assessment.
[3]Resuce medications include NSAIDS, Opiates and Other.
[4]NSAIDS include Aspirin, Diclofenac, Ibuprofen, Mefenamic acid, Naproxen, Neproxen, Neobrufen, Feminax, Anadin and Meloxicam.
[5]Opiates include Zapain, Migraleve, Oramorph, Solpadeine, Pethidine, Tramadol, Morphine, Dihydrocodeine, Co-dydramol, Codeine Phosphate, Co-codamol, MST, Tramacet and Solpadol.
[6]Other includes Paracetamol.

Table S 6 Summary of number of visits to health care professionals

	Baseline[1]		End of study[1]	
	Gabapentin n (%)	Placebo n (%)	Gabapentin n (%)	Placebo n (%)
General Practitioner				
N	152	152	111	109
0	103 (68)	118 (78)	62 (56)	62 (57)
1	28 (18)	16 (10)	16 (14)	21 (19)
2	12 (8)	10 (7)	16 (14)	11 (10)
3+	9 (6)	8 (5)	17 (16)	15 (14)
Hospital Outpatients				
N	152	152	111	109
0	139 (91)	126 (83)	89 (80)	83 (76)
1	10 (7)	22 (14)	16 (14)	20 (18)
2	2 (1)	3 (2)	2 (2)	4 (4)
3+	1 (1)	1 (1)	4 (4)	2 (2)
Practice Nurse				
N	152	152	110	109
0	142 (93)	146 (96)	99 (90)	103 (94)
1	7 (5)	6 (4)	8 (7)	3 (3)
2	1 (1)	0 (-)	1 (1)	1 (1)
3+	2 (1)	0 (-)	2 (2)	2 (2)
Physiotherapist				
N	152	152	110	109
0	147 (97)	151 (99)	107 (97)	105 (96)
1	5 (3)	1 (1)	1 (1)	0 (-)
2	0 (-)	0 (-)	1 (1)	1 (1)
3+	0 (-)	0 (-)	1 (1)	3 (3)
Other[2]				
N	151	152	110	109
0	141 (93)	143 (94)	99 (90)	97 (89)
1	5 (4)	5 (4)	6 (5)	5 (4)
2	2 (1)	2 (1)	2 (2)	3 (3)
3+	3 (2)	2 (1)	3 (3)	4 (4)

[1]Baseline-In month prior to randomization. End of study (week 16-17)-Since taking trial medication.
[2]Other includes alternative therapist, accident and emergency, occupational health, out of hour's doctor, health psychologist, pain specialist, massage therapist, research nurse, research midwife, hospital, dentist, paramedic, radiologist, eye hospital, and counsellor for cognitive behavioural therapy.

Table S 7 Adherence

	**Gabapentin**<br>(N=112)<br>n (%)	**Placebo**<br>(N=109)<br>n (%)
How frequently woman reported taking her study drug		
Never (0%)	0 (-)	2 (2)
Hardly any (1-24%)	7 (6)	1 (1)
Some (25-49%)	4 (4)	5 (5)
Most (50-74%)	10 (9)	10 (9)
Almost always (75-99%)	36 (32)	34 (31)
Every day (100%)	55 (49)	57 (52)

Figure S1 Overall median dose of study drug taken daily per week

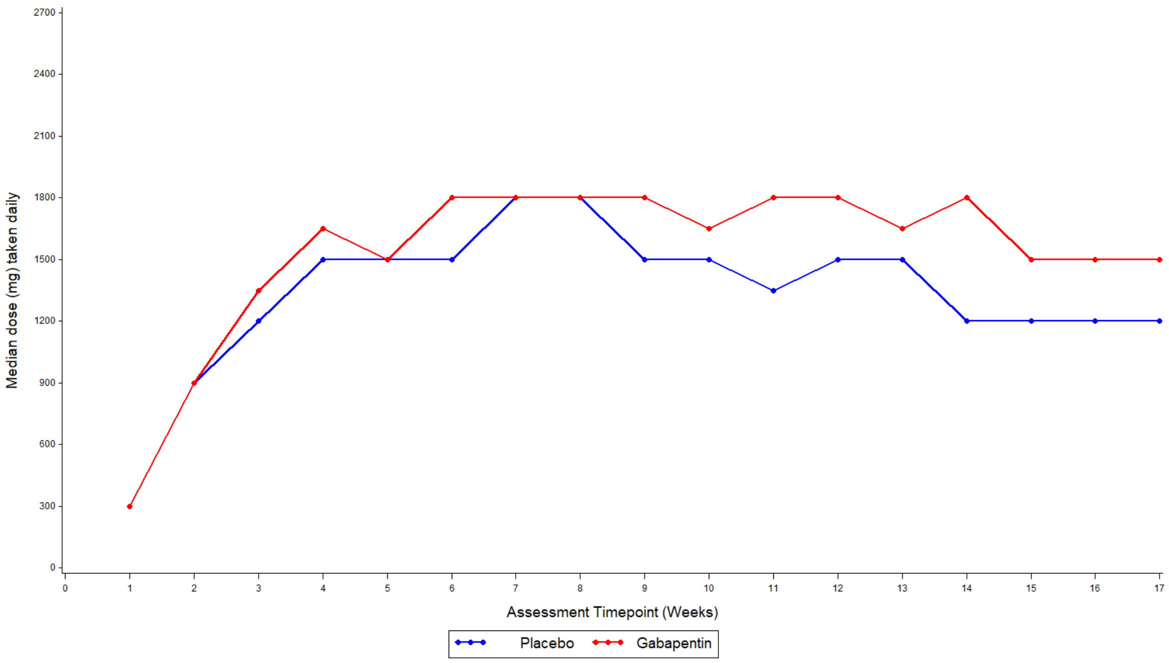

Supplement: Supplementary appendix [file mmc1.pdf]
